# Book Review

**Published:** 1893-09

**Authors:** 


*Reform in the Treatment of the Insane.* By D. Hack Tuke, M.D. Pp. 96. London: J. & A. Churchill. 1892.—The title of this book, no doubt quite unintentionally, is somewhat misleading, as any non-alienist member of the profession might expect to find some exposition of a newer and more enlightened treatment of the insane than even at present exists; whereas it only treats of the beginnings of the present system. The book is mainly a reprint of an address given by Dr. Hack Tuke at a meeting at the York Retreat on May 6th, 1892, to celebrate its centenary, under the better title of "A Retrospective Glance at the Early History of the Retreat, York; its Objects and Influence." This address was printed in the *Journal of Mental Science*, July, 1892. The book in addition contains an account of the "Celebration" from the *Yorkshire Herald*, as well as of the Annual Meeting of the Medico-Psychological Society, held subsequently at the Retreat in connection with the centenary on July 21st, 1892. The work shows how a more thoughtful and kindly method of care and treatment of the insane under great difficulties and much opposition was inaugurated at York by Mr. William Tuke, "a philanthropic citizen of York," and a member of the Society of Friends, who was greatly shocked and distressed at the treatment he saw carried out at York Asylum and elsewhere; when, as was commonly the case in such places at the time, the treatment was of a very barbarous, and often cruel, character. In many cases the patients were fastened with heavy chains to the wall or floor, to which frequently were attached balls or weights of many pounds. They were bedded in straw in wooden troughs, and lived in the greatest filth and dreadful pain and wretchedness. In the Retreat a more humane and gentle method was at once inaugurated. This has commonly been called the "Non-restraint System"; but it must be remembered that restraint was not abolished, only that when used, it was used with great discrimination, in a proper and simple form, and only for urgent and dangerous cases. The Retreat at York no doubt had a very wide influence in introducing the present gentle and kindly methods now in use; and also the use of a stimulating in place of a depressing medical treatment. We must not, however, forget, in giving due honour to the Tukes and Dr. Conolly, that in the West, even before 1792, Dr. Fox had been using a similar treatment. The book is an interesting account of how the Tukes founded and developed an asylum a hundred years ago, upon a more enlightened plan than was at that time the rule.

*Antiseptic Dry-air Treatment of Consumption.* By JOHN J. HARTNETT, M.D. 2nd Edition. Pp. 104. London: J. & A. CHURCHILL. 1892.—This book embodies most of the modern views with regard to the etiology, prevention, and treatment of phthisis pulmonalis. Dr. Hartnett strongly urges the breathing of air, purified by means of his antiseptic dry-air exhaler. If this air purifier is not procurable he would use a pulmonary insufflator for the insufflation of medicated air dry and compressed. He also attaches great importance to respiratory gymnastic exercise.

*The Value of Hypnotism in Chronic Alcoholism.* By C. LLOYD TUCKEY, M.D. Pp. 57. London: J. & A. CHURCHILL. 1892.—This pamphlet is a reprint of a paper read by Dr. Tuckey at the British Medical Association Meeting in Nottingham. Regarded from a scientific point of view, it is a little wordy and prolix, and perhaps hardly worth publication; but, considering the little use made of hypnotic suggestion in the treatment of chronic alcoholism, the paper will be useful in redirecting attention to a therapeutic measure of distinct service in many cases. It is obvious that this method has advantages over residence in a "retreat," in that it does not dislocate family life, and has no "tendency to make a man an incurable loafer" (to use a patient's expression), and it might well be tried before resorting to such an expedient. As might have been expected, the author finds that suggestion during hypnosis "succeeds especially in cases of acquired alcoholism without any hereditary tendency when the subject is desirous of cure but lacks the will power to take the initial step." It is, of course, readily admitted that in perhaps half the number of cases cure or amelioration by hypnotic suggestion is impossible, for if there be no desire to be cured, suggestion cannot strengthen it, and can but rarely produce such a tendency.

*Advice to Women on the Care of Health before, during and after Confinement.* By FLORENCE STACPOOLE. Pp. 134. London: CASSELL & COMPANY. 1892.—Not the least of the sufferings which a woman in her first pregnancy has to undergo is the ill-timed conversation of her acquaintances, who too often fill her mind with vivid pictures of dreadful things which are not at all likely to happen. She will be saved much distress if she will turn a deaf ear to information of this sort, and be guided by the sensible directions she will find in this booklet, which, notwithstanding the inclusion of one or two things that would have been better left out, we have no hesitation in recommending. There is an amusing misprint of "naval" cord.

*Our Sick: and How to Take Care of Them.* By FLORENCE STACPOOLE. Pp. 154. London: CASSELL & COMPANY. 1892.—The friends of a sick person are usually a long time learning to temper their affectionate devotion with intelligence, but of late years there has been ground for believing that there is considerable improvement in this respect. It is not given to everyone to employ or to endure a trained nurse. This little book is just the thing to guide into right paths those upon whom comes the work of nursing their friends.

*How to Give Gas.* By T. E. CONSTANT. Pp. 36. London: J. P. SEGG & CO. (N.D.)—This is a short and thoroughly practical account of the best mode of administering nitrous oxide by one who has evidently had experience. We cannot quite agree with the author when he says that the gasometer is now only a relic of the time when it was customary for dentists to manufacture their own gas, and that the method of administering it direct from the gas-bottle with the intervention of a bag is in every way better. Upon the whole, however, the author's suggestions and advice are thoroughly to be relied on.

*Notes on the Clinical Examination of the Blood and Excreta.* By SIDNEY COUPLAND, M.D. 3rd Edition. Pp. 64. London: H. K. LEWIS. 1892.—We are not surprised that a third edition of Dr. Coupland's excellent little work has been so soon called for. It exactly meets the needs of clinical clerks and of practitioners who have not time to refer to the larger manuals. Its size enables it to be carried in the pocket, and used for immediate reference in clinical work. In our opinion, it is by far the best work of the kind, and should be placed in the hands of every clinical clerk. We would suggest that in future editions room should be found for a scheme of fuller directions for the examination of the gastric juice by the omission of the description of Liebig's test for urea and of Dr. Johnson's picro-saccharimeter.

*Transactions of the American Surgical Association.* Vol. X. Pp. xxxii., 280. Philadelphia: WILLIAM J. DORNAN. 1892.—The present volume of these *Transactions* is fully up to the high standard of its predecessors. Among its principal contents we notice an excellent and exhaustive paper on "The Surgery of the Tongue," by Dr. N. P. Dandridge of Cincinnati, and one on "The Treatment of Fractures of the Lower End of the Humerus and Base of the Radius," by Dr. John B. Roberts of Philadelphia. Both these papers are accompanied by a full report of the discussion which followed, thus giving us the views of the leading American surgeons on these important topics. Of the other papers we can only mention one on "Upward Dislocations of the Hip," by Dr. Lewis Stimson of New York, and one on "A Report of some Interesting Cases of Cerebral Tumour," by Dr. Frederic S. Dennis of New York. All the articles are well worthy of perusal, and show much originality of thought as well as literary industry. The volume is enriched by half-a-dozen excellent illustrations.

*Notes on the Malarial Fevers met with on the River Niger, West Africa.* By W. H. CROSSE. Pp. 106. London: SIMPKIN, MARSHALL, HAMILTON, KENT & CO. LIMITED. 1892.—Mr. Crosse, who, as Principal Medical Officer of the Royal Niger Company, had many opportunities of observation, here places before the reader all the important facts connected with malarial fevers in this deadly region of West Africa. His book cannot fail to be useful not only to medical men whose duty or inclination may lead them to the West Coast, but also to the non-professional reader who may find himself far removed from medical advice. The author well describes the clinical aspects, pathology, and treatment of intermittent fever, remittent fever, and blackwater fever—the three great varieties of malarial fever met with in the Niger territory. An important and practical part of Mr. Crosse's book will be found in the precautions which he advises to be adopted by persons who are residing or who intend to reside in this part of tropical Africa. The book has neither a table of contents nor an index.

*Transactions of the Royal Academy of Medicine in Ireland.* Vol. X. Dublin: FANNIN & Co. 1892.—This volume is a record of much useful and interesting work in all departments of medicine and surgery, and is well worthy of careful study. Of the 243 Fellows as many as 45 have given contributions, which amount to fifty in number. Three papers on enteric fever and its treatment embody the most recent views on this important topic, and some recent aids to the diagnosis and treatment of diseases of the stomach are well described.

*Ophthalmic Atlas.* By FRANK HAYDON. London: DOWN BROS.—This affords a very convenient way of recording pathological conditions of the fundus by means of superimposed layers of colour. The plates represent the optic disc and an orange-red fundus, with retinal arteries and veins ramifying upon it. By careful scratching with a penknife, any part of the vessels may be obliterated without interference with the orange-red reflex. A more energetic use of the knife will expose the black layer which lies underneath, and thus pigmentation can be depicted, while deeper still is a white layer, by exposing which the sclera is represented as it appears in states of atrophied choroid.

*Influenza.* By JULIUS ALTHAUS, M.D. Second edition. Pp. xii.—407. London: LONGMANS & Co. 1892.—This truly exhaustive work has well drained the literature of the subject; the mere bibliography and index extend over 44 pages. The profession should be much indebted to the author for his careful analysis of so immense a mass, in many languages, on a subject in which everyone takes more or less interest. He proposes a theory which appears to explain satisfactorily—

1. Why patients acquire influenza;
2. Why they recover from it, either perfectly or imperfectly; and
3. Why, after having had it once, they contract it again a second or third time.

This theory supposes the existence of a poisonous albumenoid secreted by the bacillus of "la grippe," which is called *grippo-toxine*, and of an antidote formed in the serum of the patient and called *anti-grippo-toxine*. If the quantity of the latter be too small to neutralise all the toxine present, the course of the disease will be protracted, and complications will follow. What these sequels are has been further described in the *Medical Magazine* for March.

*The Middlesex Hospital. Reports of the Medical, Surgical, and Pathological Registrars for the Year* 1891. Pp. 350. London: H. K. LEWIS. 1892.—The Middlesex Hospital Reports give, as usual, a complete account of the work done in the Hospital during the previous year. The more important cases, medical and surgical, are given in greater detail. There is nothing to call especial attention to in this volume, but we would remind our readers of the mine of clinical and pathological facts which is presented to them in these Reports.

*The More Severe Forms of Lateral Curvature of the Spine.* By NOBLE SMITH. Pp. 24. London: SMITH, ELDER, & Co. 1892. —This is a description of the author's apparatus for treating severe cases of lateral curvature, illustrated by photographs of cases. Poro-plastic felt is condemned, and steel recommended as the most suitable material for making the supports. The work is evidently the result of considerable experience in this class of cases.

*Patent alias Quack Medicines.* 2 vols. By the Editor of *Hygiene*. London: BEAUMONT & Co. [N.D.]—These reprints are able and spirited attempts to "prick the wind-bag of quackery." They give much interesting information as to the composition of the numerous specifics which are sold under cover of the patent medicine stamp, and by the aid of constant advertisements. They are worthy of perusal, as showing what worthless trash mankind believes in.

*The Hygiene of the Ear.* By Dr. VINCENZO COZZOLINO. Translated from the Fifth Italian Edition by JAMES ERSKINE, M.A., M.B. Pp. viii., 52. London: BAILLIERE, TINDALL AND COX. 1892.—This little work has been already published in French, German, Swedish, Spanish, and Russian, as well as in the original Italian, and we are indebted to Dr. Erskine for now giving it to us in English. It will well repay a careful perusal, as it is full of valuable suggestions as to the management of the ear in health and in disease.

*Chart for Recording the Examination of Urine.* London: H. K. LEWIS.—This chart gives in a tabular form the normal and abnormal constituents of the urine, with a space after each in which to record the amount present in the specimen examined. If anyone requires such a chart, this would answer the purpose very well, but we do not see the practical advantages of it.

*Hygiene and Public Health.* By B. ARTHUR WHITELEGGE, M.D. Second Edition. Pp. 576. London: CASSELL & COMPANY LIMITED. 1893.—We are glad to welcome this new edition of Dr. Whitelegge's most practical manual, which contains an immense amount of information in a small compass. The chapters dealing with infectious diseases and their prevention show experience and judgment in every page, and in the statistical chapter a difficult subject has been carefully and clearly handled. This manual can be recommended for perusal to all engaged in practical public health work; but its very conciseness necessitates careful reading. The new edition has been carefully corrected and brought up to date.

*Atlas of Clinical Medicine.* Vol. II. Part II. By Byrom Bramwell, M.D. Edinburgh: Constable & Co. 1892.—This part of the Atlas continues the account of some cases of obscure cerebral disease, in which alterations in the visual fields was either the only or the most important symptom in the diagnosis. The cases are of remarkable interest, and should draw attention to the importance of investigating the visual fields as a matter of routine in intracranial disorders. A well-written essay on syphilis follows, and is illustrated by several plates. The part concludes with a timely and very practical article on Asiatic cholera. This is excellently written, is thoroughly up to date, and full information is given on the subject of treatment. Every practitioner should read the advice here given as to the details of the management and treatment of cases of cholera, and of the diarrhœa prevalent during cholera epidemics. An epitome of Dr. Haffkine's lecture on his method of protective inoculation is appended.

*The Journal of Pathology and Bacteriology.* Vol. I. No. IV. June, 1893. Edinburgh: Young J. Pentland.—Perhaps the most interesting papers in this number are those of Dr. Soltau Fenwick on "The Pathology of Acute Perforating Ulcer of the Stomach," and by Mr. Hopkins "On the Estimation of Uric Acid in the Urine." Dr. Fenwick criticises the time-honoured alleged causes of acute perforating gastric ulcer—*e.g.* embolism, thrombosis, venous obstruction, punctiform hemorrhages—and has little difficulty in shewing that it is most improbable that any one takes the least share in producing it. He then suggests that the cause is to be found in inflammation and necrosis of the solitary glands which occur in the gastric wall. The distribution of these lymphoid masses—*i.e.* more thickly studded in the pyloric region next the lesser curvature—and their gradual disappearance in later life fit in well with this theory, which, however, labours under the difficulty that acute perforating ulcer is nearly always solitary, whereas the lymphoid follicles are many in number; and we still need to find out why inflammation of a lymphoid follicle occurs. Mr. Hopkins's paper is a most valuable account of a method he has found of estimating uric acid in urine by precipitating it with ammonium chloride—a method which is far less tedious than that of Salkowski and much more accurate than Haycraft's. It appears very probable that this discovery will lead to a rapid extension of our knowledge of the part played by this body in the animal economy.

*Annual Report of the Medical Officer of Health, City and County of Bristol*, 1892.—1893.—The features of this carefully-prepared volume are, "The Special Report on Cholera Precautions" and the "Rules for Preventing the Spread of Consumption." Both are well worthy of careful study. When the isolation hospital is complete, the city medical officers will have a better opportunity of dealing with those cases which arise, and of stamping out the various zymotic diseases. Without such hospital the work of the medical officer of health must be arduous, and more or less unsatisfactory in its results.

*Physical Diagnosis.* By G. A. Gibson, M.D., and William Russell, M.D. Second edition. Pp. xvii., 383. Edinburgh: Young J. Pentland. 1893.—We cannot wonder that a second edition of this useful students' guide has been soon demanded. Its clear and concise descriptions, and its very numerous illustrations, leave little scope for criticism. We think, however, that only a fraction over two pages is scarcely adequate scope for all the important characters of sputum, and we doubt if a student would ever discover the bacillus of tubercle without further assistance than that given on page 181, where surely the composition of the stains should be given. Why is it that a double stain is always insisted on, when a single stain, either blue or red, on a clear field, answers every purpose?

*Advice to Intending Visitors to Cannes.* By H. Blanc, M.D. Pp. 42. London: J. & A. Churchill. 1893.—The author, well known from his captivity in Abyssinia, and from his researches in vaccination, gives what he believes to be an impartial account of Cannes, of the diseases which are benefited and of those which are not improved by residence there. The following summary gives a good idea of the contents of the book: "In a few words we may say that the climate of Cannes is indicated to those in good health who desire a change or wish to avoid the cold and fogs of England and of the north; it is indicated in convalescents from acute disease; for those whose overworked brain requires rest; to all those who in northern climes suffer from catarrhal affections; to the scrofulous, the lymphatic, the dyspeptic, to children and young adults affected with tubercular diseases of the bones; in certain forms of phthisis and gout; to the rheumatic; in certain chronic diseases of the kidneys; in some forms of asthma; in functional albuminuria; and in those who return to Europe after years spent in tropical lands. On the other hand, Cannes should be avoided by those suffering from certain nervous affections, by those having a tendency to hemorrhage, those suffering from acute febrile phthisis, and those who are prone to attacks of acute gout or eczema." A perusal of the book gives one the impression that Cannes must be a most delightful winter resort: happy should the visitors be, and the doctors whose good fortune it is to be with them.

